# Starting with ultrasonography decreases popliteal block performance time in inexperienced hands: a prospective randomized study

**DOI:** 10.1186/1471-2253-12-33

**Published:** 2012-12-19

**Authors:** Rita Cataldo, Massimiliano Carassiti, Fabio Costa, Matteo Martuscelli, Maria Benedetto, Francesco Cancilleri, Andrea Marinozzi, Nicolò Martinelli

**Affiliations:** 1 Department of Anesthesia and Intensive Care, Campus Bio-medico University, Via Alvaro del Portillo, 200, 00128, Rome, Italy; 2 Department of Orthopaedic and Trauma Surgery, Campus Bio-medico University, Via Alvaro del Portillo, 200, 00128, Rome, Italy; 3 Foot and Ankle Unit, Galeazzi Hospital, via Riccardo Galeazzi 4, 20161, Milan, Italy

**Keywords:** Peripheral nervous block, Hallux valgus, Resident’s education

## Abstract

**Background:**

The widespread of hallux valgus surgery in a day care setting enhanced the role of regional anaesthesia in the last few years. Sciatic nerve block at popliteal fossa has been shown to provide safe and effective analgesia. Our purpose was to compare the success rate and performance time of popliteal block during resident’s training for regional anaesthesia by using nerve stimulation (NS) or combined nerve stimulation and ultrasound (NS + US).

**Methods:**

70 adult patients undergoing hallux valgus surgery were randomly assigned to receive sciatic nerve block at popliteal fossa with US+NS or NS alone with a double injection technique for peroneal and tibial branches, respectively. Two residents experienced with nerve stimulator performed the procedures after a learning phase concerning ultrasonography. A local anaesthetic solution, containing 10 mL of 0.75% ropivacaine and 10 mL of 2% lidocaine was used: 12 mL were infiltrated close the tibial nerve, and 8mL were infiltrated close the common peroneal nerve. Block success rate, sensory block onset time, block performance time were evaluated. Recourse to general anaesthesia was considered as failure.

**Results:**

No differences were detected in success rate and onset time of sensory block between the two groups (P > 0.05). The time to block tibial nerve and the overall block time were significantly faster in US+NS group (P < 0.05).

**Conclusions:**

Ultrasound guidance for popliteal nerve block resulted in similar success rate with a faster procedure time when compared with nerve stimulator, thus providing a possible effect on resident education and operating room efficiency.

## Background

 The widespread of hallux valgus surgery in a day care setting enhanced the role of regional anesthesia in the last few years. Among the different peripheral nerve block techniques used for forefoot surgery, sciatic nerve block at popliteal fossa provides safe and effective analgesia reducing the doses of local anaesthetic, opioids and minimizing the risk of complications [1].

The role of ultrasound in performing nerve block is increasing over the more traditional technique of peripheral nerve stimulator. Compared to neurostimulation, ultrasound has been shown to improve the success rate and to reduce the block performance time. [2,3]. However, the learning curve of ultrasonographic technique may result in a prolonged time to achieve anesthesia, thus impairing the efficiency of the operating room.

The aim of this study was to compare the success rate and performance time of popliteal block during training for regional anaesthesia by using nerve stimulation (NS) or combined nerve stimulation and ultrasound (NS + US), in resident naïve to the use of US while considered experienced in NS guidance.

## Methods

After obtaining patient’s written informed consent and the approval of Ethical Committee of University Campus Bio-Medico of Rome, two groups of 35 patients, scheduled for hallux valgus correction between April 2008 and June 2009, were included in this prospective randomized study. A distal first metatarsal osteotomy (Chevron) was performed in all patients with ankle tourniquet application (250 mmHG).

The patients were randomized using sealed envelops to receive popliteal block with ultrasound guidance coupled with nerve stimulation (US+NS) or nerve stimulation alone (NS). Inclusion criteria were: 18 to 75 years of age, American Society of Anesthesiologists Physical Status classification < III and body mass index < 30. Exclusion criteria were: patients refusal to the procedure, neurologic or neuromuscular disease, acquired or congenital coagulopathy, skin infection at needle insertion site.

In all patients an intravenous peripheral access was established and routine noninvasive blood pressure, electrocardiogram and pulse oximetry monitors were applied. Premedication, consisting in Midazolam 0.03 mg/Kg, was administered before block placement.

Patients were positioned prone with the feet protruding off the bed, in order to detect myoclonies in distribution fields of both common peroneal and tibial nerves. 

Blocks were performed by two residents with prior experience in regional anesthesia using nerve stimulator but novice to ultrasound and novice to popliteal block, as well. The residents were attending the second year of Residency in Anesthesia and Intensive Care and had already performed forty-five and fifty-one peripheral nerve blocks with NS, which included axillary brachial plexus block, interscalenic brachial plexus block, transgluteal sciatic nerve block and inguinal femoral nerve block. Before starting the study, residents received didactic instruction in the anatomy of popliteal fossa, as well as a two days training in ultrasound-guided nerve localization for regional anesthesia and for sciatic nerve localization at popliteal fossa, particularly. Furthermore, two senior staff of the Anaesthesiology department provided bedside teaching in recognizing surface landmarks and needle placement. Ten preliminary procedures were performed by each resident (five with NS and five with US+NS) assisted by the senior staff. During the study, the residents only were the operators. Skin marking, ultrasonographic image interpretation, decisions on injection were made on their own. The senior staff was available in case of difficulties. Landmarks on the poplital fossa were drawn: a line was signed across the popliteal crease, extending between the tendons of the biceps femoris (lateral) and the semitendinosus muscles (medial). The needle puncture site was identified by calculating 7 cm proximal to the popliteal crease on the midline axis and then moving 1 cm lateral to the same line. Ultrasound scanning consisted in positioning a linear 7,5-12 Mhz probe 7 cm above the popliteal crease. A sterile adhesive tape and a sterile ultrasound jelly were used. The probe was aligned to detect vessels and nerves in short axis. An attempt to visualize the sciatic nerve division has been performed in all cases. However, considering that sciatic nerve division lies at 3–5 cm depth, its visualization is sometimes difficult and needs more experience. The probe was then moved distally, in order to detect the sciatic nerve branches. The residents were allowed to inject the local anaesthetic solution where the best ultrasound nervous scanning was obtained to identify clearly the two sciatic branches. The needle was inserted and advanced “in plane” with the probe and once gained the targeted nerve, the nerve stimulator was switched on and the stimulating current increased up to obtain a motor response. The nerve stimulation was just used as a confirm of the right US identification of targeted nerve. Then it was switched off and the procedure was accomplished only by ultrasound guidance. After skin disinfection with 2% chlorhexidine in 70% isopropyl alcohol solution, a 20 mL of standardized local anaesthetic solution, containing 10 mL of 0.75% ropivacaine and 10 mL of 2% lidocaine without epinephrine was used. 12 mL were infiltrated close the tibial nerve, and 8mL were infiltrated close the common peroneal nerve.

In the NS group, a 21-gauge insulated needle (Stimuplex, B. Braun USA, Bethlehem, PA) was inserted and advanced until a motor response elicitation (plantarflexion for tibial nerve and dorsiflexion for common peroneal nerve) was detected using a current of 1 mA at a frequency of 2 Hz in 100 μsec. The perineural position was considered optimal when a minimal intensity stimulating current (0.4 mA) still evocated foot movements in the tibial and common peroneal nerve distribution fields. Then, after negative aspiration, the local anaesthetic was slowly administered.

During block performance the following times were registered: T1= time to perform tibial nerve block (time interval between the needle insertion and complete local anaesthetic administration close tibial nerve); T2= time to perform common peroneal nerve block (time interval between tibial block and complete local anaesthetic injection close common peroneal nerve). At the end of the procedure an examiner, blinded to patient group allocation, assessed the progress of sensory and motor blockade at 5 min intervals up to 30 min. Sensory function was tested by application of a small ice pack on dorsal and plantar surface of the foot. The quality of sensory block was rated as: 2= normal ice sensation; 1= partial loss of ice sensation; 0= complete loss of ice sensation. Onset time of sensory block was assessed as follow: T3= onset time of tibial nerve block (time interval between the end of anaesthetic administration close tibial nerve and onset of sensory block in its distribution territory); T4= onset time of common peroneal nerve block (time interval between the end of anaesthetic administration close common peroneal nerve and onset of sensory block in its distribution territory). The definition of sensory block was a loss of ice sensation, with a score of 0. Plantar flexion and dorsiflexion were both assessed to test motor function of tibial and common peroneal nerves, respectively. Motor function was rated as: 2= full muscle strength; 1= strength reduced from baseline; 0= complete loss of motor response. Motor blockade was considered to be complete when a score of 0 was reached for both tibial and common peroneal nerves dependent movements. Block success was defined as loss of ice sensation within 30 min after local anaesthetic injection and confirmed by pain absence at surgical incision or intraoperatively. Otherwise, block failure was defined as pain at skin incision or during surgical procedure requiring conversion to general anesthesia or need of intraoperative local injection of anesthetic. The number of needle punctures was also recorded for each group.

A complete motor block was considered not necessary to get the patient in the surgical theatre. As soon as the Ice test became positive we allow the access of patient in the operating room, also considering the times for patient monitoring and positioning.

Block performance time weas registered by the senior staff who was not blind to the procedure. Once the nervous block was performed, senior staff and residents left the pre-anaesthesia room and let a blind observer to have access and monitor the onset and progression of sensory-motor block. The same blind observer collected the patient satisfaction level in the post-operative period.

Patients were asked to subjectively evaluate the satisfaction level at the end of block performance with the following categories: “very satisfied”, “satisfied” and “not satisfied”.

Based on prior works, we hypothesized that ultrasound guidance combined with neuro-stimulation could have increased of 30% the success rate of tibial and common peroneal nerve block at the popliteal fossa in comparison with neuro-stimulation alone during resident education [2]. A sample size estimation using a two-tailed test with a type 1 error of 0.05 and power of 80% determined that a significant difference in the success rate of popliteal block would have been detected with a minimum of 32 patients for each group. In order to face possible drop-out 35 patients were enrolled for each group.

Analysis were performed using SPSS 13.0 for Windows (SPSS, Chicago, IL). Data are presented as mean ± standard deviation (SD) for continuous variables, and number or % of total patients in each group for discrete and categorical variables. Un-paired Student’s *t*-test was used for comparison of the continuous variables between the two study groups and the non-parametric Mann–Whitney test was used to compare the nonnormal distributed variables. The clinical success rate for both groups was compared with the *χ*^2^ test. Statistical significance was set at 0.05.

## Results

Seventy patients were enrolled in the present study. No statistically significant differences were founded between the two groups in terms of age, weight, height, BMI and gender (Table 1). No patients were excluded from both study groups. In NS + US group direct visualization of tibial nerve was possible in all cases but common peroneal nerve was directly visualized in 31 patients. In four patients common peroneal nerve visualization was considered fair, but a typical motor response to nerve stimulator (dorsiflexion and eversion) allowed to complete the block. However two patients for each group needed conversion to general anesthesia because of pain at skin incision or during surgical procedure. Three patients in the US + NS group and two patients in the NS group received additional sedation with propofol due to intraoperative anxiety, so that they were not considered as block failure.

**Table 1:** Demographic characteristics of the study groups

	**US + NS (n = 35)**	**NS (n= 35)**	**P**
**Age (years)**	57.06 ± 9.28	53.94 ± 10.65	0.7
**Weight (kg)**	66.46 ± 7.32	65.91 ± 8.20	0.2
**Height (cm)**	161.83 ± 6.38	161.97 ± 6.02	0.7
**Gender (Female/Male)**	31/4	30/5	0.7
**BMI**	25.20 ± 3.10	25.06 ± 3.77	0.8

Data are presented as mean ± SD, except for gender (n. of patients).

Procedure performance time, onset time of sensory block and needle insertion number are listed in Table 2. The time to perform tibial nerve block and the overall block time significantly differed between the two groups (P < 0.05). However, the time to block common peroneal nerve did not show significant differences between the two study groups (P > 0.05). The onset time of sensory block for tibial and common peroneal nerves was similar for each type of block performance (P > 0.05). At 30 min a complete motor blockade was obtained in 30 patients (85.7%) in US + NS group and in 22 patients (62.8%) in the NS group, thus revealing a statistically significant difference between the two groups (P < 0.05). Block success at 30 min showed the same rate in all patients (94.2%). No early complications were observed during block performance, except for an accidental puncture of popliteal artery in NS group not related to local or systemic toxicity.

**Table 2:** Comparison of block features between the study groups

	**US + NS (n = 35)**	**NS (n = 35)**	**P**
**Overall block time (sec)**	379.97 ± 145.52	461.71 ± 152.57	0.02
**Time to perform tibial block (sec)**	138.86 ± 77.75	180.86 ± 75.31	0.02
**Time to perform common peroneal block (sec)**	243.97 ± 123.32	280.86 ± 118.67	0.2
**Total motor block at 30 min (%)**	30/35 (85.7)	22/35 (62.8)	0.029
**Block success at 30 min (%)**	33/35 (94.2)	33/35 (94.2)	> 0.05
**Onset time tibial block (min)**	17.4 ± 5.0	18.5 ± 6.3	0.6
**Onset time common peroneal block (min)**	17.8 ± 7.6	19.2 ± 6.2	0.3
**No. of needle insertions**	2.2 ± 0.9	1.6 ± 0.73	0.004

Data are presented as mean ± SD or no. and % of total patients.

The overall satisfaction level resulted higher in the US + NS group (P < 0.05). In US + NS group twenty-four patients (68.5%) were very satisfied with the procedure, nine (25.71%) were satisfied and two patients (5.71%) were not satisfied. In NS group fifteen patients were very satisfied (42.85%), twelve were satisfied (34.28%), eight patients were not satisfied (22.85%).

## Discussion

The authors supporting the role of ultrasound guidance technique for peripheral nerve block state that ultrasound can provide a better nerve visualization resulting in a more accurate block with higher success rate, lower incidence of neuropathy or accidental vessel puncture, shorter block performance time, shorter block onset time, higher patient satisfaction in comparison with neurostimulation [4-7]. However concerns about actual advantages of ultrasound guidance still exist. Perlas et al. showed that ultrasound guidance improves the success rate of popliteal sciatic nerve block in comparison with neurostimulation (89.2% vs 60.6%) [2]. Danelli et al. [8] reported a similar success rate for either procedures using a multiple injection technique (100% vs 82%). Casati et al. [9] demonstrated that in experienced hands, both techniques have comparable success rate and complication incidence in peripheral nervous block. However, few studies concerning ultrasound impact on the education of anaesthesiology residents in performing peripheral nerve block have been published [10]. Given the low experience of residents, a higher incidence of such complications as neuropathy or vessel puncture should be taken into account.

Previous studies demonstrated that the use of US by inexperienced anaesthetist residents resulted in a higher rate of successful blocks for infraclaviclular, axillary, interscalene, femoral and popliteal fossa blocks [10,11]. However, it is not easy to compare these results because of the variability in skills and experience to perform peripheral nerve blocks and no prospective randomized studies comparing the use of ultrasound to neurostimolation during training in regional anaesthesia have been published.

The results obtained in this prospective randomized study suggest that combined ultrasound and neurostimolator guidances did not increase successful block rate of tibial and common peroneal nerves at the popliteal fossa during the learning phase of the procedure in supervised residents. Both technique showed high level of efficacy (94.2%) and no significant difference was detected with respect to the onset of sensory block of both branches of the sciatic nerve.

The success rate at thirty minutes was similar in both groups using a double-stimulation technique and comparable with previous studies. Furthermore, no differences in terms of complications such as neuropathy were noted.

Although an unwanted blood vessel puncture occurred in the NS group, our findings do not allow to draw final conclusions regarding the effect of ultrasound on the incidence of complications in popliteal block performed by inexperienced residents. Furthermore, given the extremely low incidence of serious side effects in performing popliteal block (i.e. neuropathy or systemic toxicity), a much larger sample size should have been enrolled to attempt determining whether the ultrasound guidance actually decreases the incidence of complications [12].

Previous studies examining block procedure time reported varying results. Dufour et al. [13] reported that block procedure time did not differ significantly between combined ultrasound and neurostimulation vs neurostimulation alone with a double injection technique (304 sec vs 261 sec). Danelli et al. [8] demonstrated that ultrasound guidance was able to reduce the procedure time with a multiple injection technique when compared with neurostimolation (2 min vs 5 min). Analysis of times needed to perform popliteal block in our study showed a significant difference between the two groups in the overall time and time to block tibial nerve, which resulted faster in US+NS group (P= 0.02). However, ultrasound guidance did not achieve a faster time to block common peroneal nerve in comparison with neurostimulation alone (P= 0.2), since common peroneal nerve has small calibre and low echogenicity. Furthermore, a higher satisfaction level was detected in US + NS group, probably related to the shorter block time. Interestingly, in US + NS group, despite a shorter block time, a higher number of needle insertions was noted. The explanation to this finding is that residents were taught to execute repeated attempts in order to obtain an optimal perineural needle tip position.

Ultrasonography has been advocated to improve peripheral nerve block quality due to direct visualization of the anatomic structures and local anaesthetic, with faster onset and lower doses of local anaesthetic [4,13-16]. However our results are in agreement with Danelli et al. [8] who showed similar success rate and sensory onset time block with ultrasonography when compared with neurostimulation using a posterior approach for popliteal block with a double-injection technique.

We acknowledge that this study has some limitations. We did not consider the experience degree of neurostimulation task and the learning curve of ultrasound in anaesthesiology residents with possible risk of bias in data interpretation. Great differences in cognitive and perceptual abilities may have lead to a wide scatter of success rates or complications. The number of residents participating to the present study was relatively small and a large enrolment of “slow learners” could have changed the results dramatically.

The cutaneous innervation of the distal medial metatarsal region can be supplied by saphenous nerve. In this case it is difficult to discern between a failed block or a false negative. In a recent paper, Sala-Blanch et al. reported excellent results performing a popliteal block with ultrasound guidance using a single-injection technique at the sciatic division. The authors did not perform a saphenous block [17]. We believe that a skin incision at the first metatarso-phalangeal joint can be managed with a popliteal block. However, further studies are necessary to clarify this topic.

## Conclusions

This prospective randomized study demonstrated that combined ultrasound guidance with neurostimulation for popliteal nerve block resulted in similar success rate and faster procedure time when compared with neurostimulation alone in inexperienced hands, thus providing a possible effect on resident education and operating room efficiency. Furthermore, we believe that visualizing anatomy with ultrasound can be an useful learning experience for anesthesia resident in order to gain the best confidence and a “picture” of the targeted region.

## Competing interests

The authors declare no competing interests in publication of this article. No funding has been received for the study.

## Authors’ contributions

RC, MC and FC conceived the study and participated in its design and coordination and helped to draft the manuscript. MM and MB performed the peripheral nerve block and collected the results. NM performed the review of the literature, wrote the initial draft and performed the statistical analyses. AM and FC performed surgery and drafted the manuscript. All authors read and approved the final manuscript.

## Pre-publication history

The pre-publication history for this paper can be accessed here:

http://www.biomedcentral.com/1471-2253/12/33/prepub
